# Maintenance repetitive transcranial magnetic stimulation (rTMS) therapy for treatment-resistant depression: a study protocol of a multisite, prospective, non-randomized longitudinal study

**DOI:** 10.1186/s12888-023-04944-0

**Published:** 2023-06-16

**Authors:** Ryuichi Yamazaki, Yuki Matsuda, Mari Oba, Hideki Oi, Shinsuke Kito

**Affiliations:** 1 Department of Psychiatry, The Jikei University School of Medicine, Tokyo, Japan; 2 Department of Clinical Data Science, Clinical Research and Education Premotion Division, National Center of Neurology and Psychiatry, Tokyo, Japan; 3 Department of Psychiatry, National Center Hospital, National Center of Neurology and Psychiatry, 4-1-1 Ogawahigashi-Machi, Kodaira-Shi, Tokyo, 1878551 Japan; 4 Neuromodulation Therapy and Research Center, National Center Hospital, National Center of Neurology and Psychiatry, Tokyo, Japan

**Keywords:** Repetitive transcranial magnetic stimulation, Maintenance therapy, Major depressive disorder, Relapse prevention

## Abstract

**Background:**

Repetitive transcranial magnetic stimulation (rTMS) is a widely used treatment for major depressive disorder (MDD), and its effectiveness in preventing relapse/recurrence of MDD has been explored. Although few small sample controlled studies exist, the protocols of maintenance rTMS therapy were heterogeneous and evidence of its effectiveness is not sufficient. Thus, this study aims to evaluate whether maintenance rTMS is effective in maintaining the treatment response in patients with MDD with a large sample size and feasible study design.

**Methods:**

In this multicenter open-labelled parallel-group trial we plan to recruit 300 patients with MDD who have responded or remitted to acute rTMS therapy. Participants would be classified into two groups according to their preference; the maintenance rTMS and pharmacotherapy group, and the pharmacotherapy only group. The protocol of maintenance rTMS therapy is once a week for the first six months and once biweekly for the second six months. The primary outcome is the relapse/recurrence rates during 12 months following enrollment. Other measures of depressive symptoms and recurrence/relapse rates at different time points are the secondary outcomes. The primary analysis is the between-group comparison adjusted for background factors using a logistic regression model. We will perform the group comparison with inverse probability of treatment weighting as the sensitivity analysis to ensure the comparability of the two groups.

**Discussion:**

We hypothesize that maintenance rTMS therapy could be an effective and safe treatment for preventing depressive relapse/recurrence. Considering the limitation of potential bias owing to the study design, we plan to use statistical approaches and external data to avoid overestimation of the efficacy.

**Trial registration:**

Japan Registry of Clinical Trials, ID: jRCT1032220048. Registered 1 May 2022.

**Supplementary Information:**

The online version contains supplementary material available at 10.1186/s12888-023-04944-0.

## Introduction

Major depressive disorder (MDD) is a chronic disorder that is characterized by frequent relapses and recurrences [1]. It is therefore imperative to develop a treatment strategy for maintenance therapy to reduce relapse/recurrence. Although maintenance therapy is commonly provided by pharmacotherapy, approximately half of patients reportedly experience relapse/recurrence even with successfully continued pharmacotherapy [2–4]. Structured psychotherapy, such as cognitive-behavioral therapy and brain stimulation therapy, such as electroconvulsive therapy, have also been explored for their efficacy in preventing relapse/recurrence [5–8]. The recurrence rate has been reported to improve with maintenance therapy using these non-pharmacological therapies in combination with pharmacotherapy, compared to maintenance therapy using pharmacotherapy alone [9–11]. However, considering the limited number of patients for whom these non-pharmacological therapies are indicated and the still high relapse/recurrence rate with the combination therapy, the current treatment options do not fully meet the needs of the patients with MDD.

Repetitive transcranial magnetic stimulation (rTMS) as a brain stimulation therapy is recommended for patients with MDD, especially for those who do not respond to pharmacotherapies [12, 13]. The effectiveness of rTMS therapy in the acute phase has already been demonstrated [14–17]. Recently, the possibility of reducing recurrence/relapse by continuing rTMS therapy in the maintenance phase has been investigated [18–28]. A meta-analysis of the long-term effects of acute rTMS in 732 depressed patients in 18 studies found that the proportion of patients who met the treatment response criteria 6 months later was 61.1% (95% confidence interval: 49.8–71.3%) in the group with and 38.5% (21.9–58.3%) in the group without maintenance rTMS [29]. Although the results seem favorable for maintenance rTMS therapy, owing to the small sample size and the heterogeneity of each study, no definite conclusions can be established concerning the efficacy of maintenance rTMS therapy. Considering the sample size, having a large sample size is challenging in a long-term study, such as maintenance therapy, owing to dropouts and other reasons; thus, it is necessary to adopt a feasible design that facilitates participation in the study. Considering heterogeneity, significant differences, especially in the schedule of maintenance therapy, including weekly (or bi-weekly) rTMS versus clustered rTMS, exist. Prior randomized controlled trials have demonstrated a certain level of effectiveness of clustered rTMS as maintenance therapy, with the advantage of reducing the number of hospital visits [30]; however, concerns remain about adverse events, since a greater amount of stimulation is performed in a shorter period of time. Although some case reports and small sample studies exist [18, 28, 31], the efficacy and safety of maintenance treatment with weekly or bi-weekly rTMS therapy remain unknown.

We hypothesized that a weekly or biweekly maintenance rTMS therapy schedule could be effective in preventing depressive relapse/recurrence. Thus, the main objective of this study was to evaluate the efficacy and safety of 12 months of maintenance rTMS therapy in patients with moderate to severe treatment-resistant MDD who respond to acute rTMS therapy with a feasible study design.

## Materials and methods

### Study overview

**Fig. 1:**
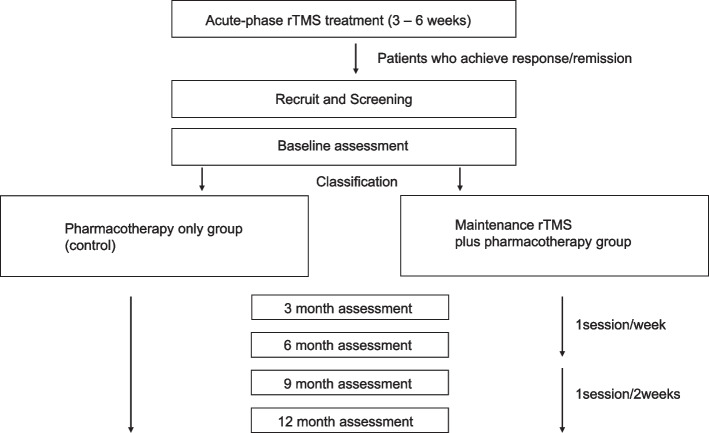
Flowchart of the study. rTMS: repetitive transcranial magnetic stimulation

This study is a multisite, prospective, non-randomized longitudinal trial, recruiting 300 participants, who will be divided into two groups: (I) the maintenance rTMS plus pharmacotherapy group and (II) the pharmacotherapy-only group. The overview of the study is shown in Fig. 1. Only patients who achieve a therapeutic response to acute rTMS therapy will be included in the study. In the maintenance rTMS plus pharmacotherapy group, patients will receive weekly treatments for 6 months, followed by bi-weekly treatments for an additional 6 months, totaling 12 months of maintenance rTMS therapy (maximum 40 sessions). During the maintenance period, the patient will continue with a stable dose of pharmacotherapy. In the pharmacotherapy-only group, patients will receive no intervention other than a continuation of concomitant pharmacotherapy and receive an evaluation. Assessments will be made at 3, 6, 9, and 12 months after the start of maintenance therapy and at the end of study participation. Random allocation to both the groups will not be performed and the patients will be classified to a group (I) or (II) according to their preferences. Owing to the long duration of maintenance therapy immediately following acute treatment in this study, conducting a double-blind, randomized, sham-controlled, parallel-group study was deemed challenging in terms of subject participation and blinding validity.

The National Center of Neurology and Psychiatry Clinical Research Review Board approved the study protocol (approval number: CR21-004) following the principles of the Declaration of Helsinki. The approval encompasses all participating centers in accordance with the Clinical Trials Act in Japan. The trial has been registered in the Japan Registry of Clinical Trials (ID: jRCT1032220048, final update is protocol version 2.0, 1 May 2022). Independent quality monitoring of the trial will be performed in order to ensure the quality of the study. Written informed consent will be obtained from all the participants before beginning the study.

### Participants

#### Inclusion criteria

Participants with MDD above the age of 18 years who responded or remitted to 3–6 weeks of acute rTMS therapy will be included. In Japan, rTMS is indicated for patients who have failed to respond to at least one or more antidepressants; thus, these patients will be included in this study. All the participants must understand and be willing to adhere to the 12-month maintenance therapy schedule. The researchers will obtain informed consent from the participants who meet the criteria.

#### Exclusion criteria

Possible pregnancy, significant suicidal ideation (suicide item score of Hamilton Rating Scale for Depression 17 item (HAMD-17) ≥ 3), and severe side effects from acute rTMS therapy are the exclusion criteria for this study. Patients with an intracranial implant (e.g., cochlear implants, magnetic clips, stimulators, such as deep brain stimulators) or other metal objects near the stimulation site (e.g., cardiac pacemakers) are contraindicated for rTMS therapy and therefore, patients with these conditions are not eligible for this study.

#### Power and sample size calculation

In the previous meta-analysis of maintenance rTMS therapy, the response rates at 3 and 6 months in the group with and without maintenance rTMS therapy were 76.2% and 61.1%, and 56.1% and 38.5%, respectively [29]. Applying the Weibull distribution to the 3- and 6-month response rates, the 12-month response rate was estimated to be 40.9% for the maintenance rTMS group and 20.7% for the no maintenance rTMS group, with an estimated difference of 20%. Considering the relapse and recurrence rates, the previous randomized controlled trial of maintenance therapy in patients who responded to pharmacotherapy reported 44.4% in the pharmacotherapy-only group and 24.2% in the rTMS maintenance therapy group, with a 20.2% difference between the groups [30]. Based on these previous studies, we estimated that the relapse/recurrence rate without maintenance rTMS was 45–60%, and the add-on effect of maintenance rTMS was 20%. To detect a 20% additional effect of maintenance therapy, the required sample size varies from 109 to 130 patients for a 45 ~ 60% relapse/recurrence rate in the pharmacotherapy-only group (chi-squared test with two-sided alpha = 0.05 and power = 80%). Since this study plans to conduct a multivariable analysis adjusting for the background factors, the power was set as high as 90%. Assuming that the proportion of participants who drop out without any evaluation of efficacy is 15%, the required number of participants would be 150 in each group.

From the viewpoint of detection of rare adverse events in the maintenance rTMS group, with 150 participants, at least one event with a 2% probability with a 95% chance is expected. Therefore, based on the perspective of comparing the efficacy and collecting safety information, the target number of study participants was set at 150 in each group, with a total of 300 participants.

### RTMS protocol

All the rTMS treatment will be performed using the NeuroStar TMS system (Neuronetics, Inc., Malvern, PA, USA) at a 120% resting motor threshold over the left prefrontal cortex at 10 Hz, with a 4-s stimulation time, 11-s or 26-s inter-stimulation interval, and 3000 pulses/day. The left prefrontal cortex stimulation site was determined by advancing the coil 5.5 cm anterior to the motor threshold location. In the event of problems caused by rTMS therapy stimulation, such as headache, move the coil within 0.5 cm of the stimulation site or rotate it -5 to + 5 degrees around the coil intersection to alleviate symptoms. Participants will be excluded from the intervention if they exhibit unacceptable adverse reactions, are diagnosed with mania or hypomania, or exhibit symptoms that would preclude them from continuing with the trial. Therapists will be in close contact with the participants to increase adherence to the intervention.

Acute-phase rTMS treatment prior to inclusion in this study will be performed as per usual practice in accordance with the Japanese guidelines for the appropriate use of rTMS [32]. Treatment will be administered once daily for up to five days a week, for a maximum duration of six weeks. If patients meet the remission criteria or conversely do not obtain therapeutic effect at the end of three weeks of the treatment, rTMS treatment will be discontinued or gradually tapered off over the course of an additional three weeks. Alternatively, treatment will be continued for 6 weeks. The patients will be administered a stable dose of psychotropic medications during the treatment period.

### Covariates and outcomes

An overview of the schedule of assessments is shown in Table 1. To ensure the reliability of the assessment, the HAMD-17 and Montgomery–Åsberg Depression Rating Scale (MADRS) will be conducted by clinical or licensed psychologists who have experience in clinical trials. Before the start of the study, we will confirm the agreement of the ratings by using a simulated patient using video clips. In principle, the same evaluator will conduct the evaluation for each participant. In this study, the response is defined as a decrease of 50% or more in the total HAMD-17 score compared to that before the acute phase of rTMS therapy. Remission is defined as a HAMD-17 total score ≤ 7. Relapse/recurrence is defined as a total HAMD-17 score ≥ 14. Cohen's d will be calculated to evaluate the magnitude of the treatment effect for each outcome.

**Table 1:** Assessment schedule

	Screening period	Maintenance therapy period				
	1 week	12 months				
	-1 week	0 month	3 month	6 month	9 month	12 month
Screening	◯					
Informed consent	◯					
Patients back ground	◯					
Maintenance rTMS		—△ →	—△ →	—△ →	—△ →	—△ →
Concomitant medications use		★	★	★	★	★
Adverse events		★	★	★	★	★
HAMD-17		◯	◯	◯	◯	◯
MADRS		◯	◯	◯	◯	◯
QIDS		◯	◯	◯	◯	◯
PHQ-9		◯	◯	◯	◯	◯

*HAMD* Hamilton Rating Scale for Depression, *MADRS* Montgomery–Åsberg Depression Rating Scale, *QIDS* Quick Inventory of Depressive Symptomatology, *PHQ-9* Patient Health Questionnaire-9

△: only for maintenance rTMS plus pharmacotherapy group

★: concomitant medication use and adverse events are assessed at each treatment session or outpatient visit

- Primary outcome
  - Relapse / recurrence rates during 12 months after enrollment
- Secondary outcome measures
  - Relapse / recurrence rates at 3, 6, and 9 months after enrollment (Those who have met the criteria for relapse/recurrence prior to each evaluation period will be treated as relapse/recurrence thereafter.
  - Response rate at 3, 6, 9, and 12 months after enrollment
  - Remission rates at 3, 6, 9, and 12 months after enrollment
  - Changes in the HAMD-17, MADRS, Quick Inventory of Depressive Symptomatology (QIDS), and Patient Health Questionnaire-9 (PHQ-9) scores since enrollment
  - Time to relapse/recurrence after enrollment
  - Adverse events and equipment failure (headache, pain or irritation at the site of stimulation, manic and hypomanic episodes that meet the diagnostic criteria of Diagnostic and Statistical Manual of Mental Disorders, 5th edition (DSM-5), suicide ideation, seizure, etc.)
- Other covariates
  - Demographic characteristics and clinical observation information (information obtained from interviews and medical records, including sex, age, comorbidities, and duration of illness)
  - Reasons for treatment selection (confirm reasons for choosing to do or not to do maintenance rTMS therapy in multiple-choice and free-text format)

### Safety monitoring

An adverse event is defined as any unfavorable medical event that occurs in a participant. A physician will examine the participant at the end of each stimulation and evaluate the adverse events. All the adverse events reported spontaneously by the participant or observed by the research team will be recorded. All the adverse events will be judged based on the intensity and their relation with the investigational product. Serious adverse events are defined as life-threatening events that require hospitalization, result in persistent or significant disability or incapacity, are a congenital anomaly or birth defect, or have a significant impact on the safety of the participant. In the event of serious health problems resulting from participation in this research, the participant may receive compensation benefits from the clinical research insurance coverage provided by the research sponsor.

### Data collection and data management

All the evaluations will be conducted by experienced psychologists. After the data are collected, all the data in the paper files will be transcribed to the Electronic Data Capture system (e Clinical Base; Translational Research Center for Medical Innovation, Kobe, Japan), which is a secure system designed for storage of personal and patient data. The data will be sent to independent data managers to assess whether the data were collected properly, focusing on the status of consent acquisition, eligibility of the participants, evaluation items, and confirmation of drop-out/terminated cases. These data managers will also oversee and review the progress of the trial. If a participant withdraws their consent, they will be dismissed from the study. At the same time, we will record the dropout rate, and the number of participants with adverse events requiring treatment. The Efficacy and Safety Assessment Committee, whose members are independent of the research, will check and assess whether the trial is conducted safely and properly, and will also decide whether to stop the trial if any severe adverse events or protocol violations occur. In addition, an on-site data monitor will conduct monitoring to ensure that the trial is performed properly, data are properly recorded, and data reliability is ensured. Audits will be also conducted independent of the researcher and sponsor. If we conduct any necessary protocol modifications, we will report them to the Clinical Research Review Board, and to the registration site of the Japan Registry of Clinical Trials website. The results of this study will be anonymized and published in relevant academic conferences and journals.

### Statistical analysis

The efficacy and safety analysis will be performed in the full analysis set, which includes all the patients who have undergone the study treatment, including those who have discontinued treatment. Missing values will not be imputed. All the reported *p*-values will be two-sided, and values of *p* < 0.05 are considered statistically significant. Data analyses will be performed using SAS (version 9.4 or later) or R (version 3.6 or later).

Baseline characteristics will be summarized using descriptive statistics according to the enrolled group. Those will be compared using chi-square tests for categorical variables and t-tests for continuous variables between groups.

#### Primary outcome

The relapse/recurrence rate will be calculated as the proportion of the patients who experienced relapse/recurrence during 12 months in each group. The odds ratio and 95% confidence interval (95%CI) will be estimated using a multivariable logistic regression model. The following covariates will be used as explanatory variables: age, sex, number of depressive episodes, duration of current depressive episode, concomitant medication, and status at enrollment (response/remission), and others to be determined prior to data fixation.

#### Secondary and other outcomes

As for the primary outcome, the rates and between-group differences, and adjusted odds ratio (95% CI) will be estimated for the relapse/relapse rate, response rate, and remission rate at 3, 6, and 9 months after enrollment, respectively. Kaplan–Meier curves will be constructed for the duration of the sustained treatment response and sustained remission, and the log-rank test and Cox proportional hazards models will be performed for the between-group comparisons.

The change in the total scores of the HAMD-17, MADRS, QIDS, and PHQ-9 from the baseline (before the introduction of maintenance therapy) will be analyzed using a random-effects model with group, baseline total score, age, sex, time point, and interaction between the time point and the group as explanatory variables and individual as random effect, for all the data at 3, 6, 9, and 12 months.

Frequency distributions of the worst severity of the adverse events and equipment failure will be summarized. Treatment-related adverse events, serious adverse events, and discontinuations owing to adverse events will also be summarized.

### Historical control

The primary outcome will also be compared to the 12-month outcomes of the post-marketing surveillance of the NeuroStar TMS system in Japan. The surveillance will include the 300 participants with MDD who received 3 to 6 weeks of acute-phase rTMS therapy in the usual clinical setting and had their prognosis with usual maintenance therapy, which consisted primarily of pharmacotherapy, evaluated.

### Sensitivity analysis

As a sensitivity analysis, we will analyze the primary outcome with inverse probability of treatment weighting (IPTW) to ensure the comparability between the maintenance rTMS plus pharmacotherapy and the pharmacotherapy-only groups. The propensity score will be calculated using the covariates listed above (primary outcome section). After weighting, the covariate balance between the groups will be assessed using absolute standardized difference. Moreover, we would perform inverse probability of censoring weighted method to consider the bias attributed to the informative missing and dropout data.

## Discussion

The main goal of this study is to clarify the efficacy and safety of maintenance rTMS in patients with treatment-resistant but rTMS-effective MDD. To the best of our knowledge, this study will be the first large-sample study to evaluate the effectiveness of weekly/bi-weekly maintenance rTMS in these patients.

The study employs a maintenance therapy protocol in which rTMS will be administered once a week for the first 6 months and then once every 2 weeks thereafter. We have already conducted a preliminary study of maintenance therapy with this protocol in two patients with treatment-resistant depression and reported that remission was successfully maintained [31]. Other previous studies have similarly employed a protocol of gradual tapering from acute treatment [18, 28]. This is reasonable considering the high incidence of relapse and relapse in the period of 6 months following the completion of acute-phase rTMS therapy [26]. However, it has been suggested that rTMS less frequently than once biweekly may not result in clinically meaningful effects [33]; thus, the protocol in this study was to maintain the pace of once biweekly during the latter half of the maintenance therapy phase.

Previous studies have reported other maintenance therapy protocols, such as the reintroduction of rTMS when symptoms worsen, or clustered rTMS, in which rTMS is administered multiple times a day for several consecutive days within a month [21, 22, 26, 30]. The reintroduction strategy may reduce the number of hospital visits, but has the disadvantage of requiring more sessions of treatment during recurrence/relapse [21, 26]. Clustered rTMS can also reduce the number of hospital visits; however, it has the disadvantage of increasing treatment time owing to multiple sessions of rTMS per day and deviating from the labeled use of the Neurostar TMS system [22, 30]. The maintenance rTMS protocol of weekly or bi-weekly rTMS may increase the frequency of hospital visits, but the total number of pulses per day is the same as the acute treatment and may increase safety and tolerability; thus, we adopted this maintenance protocol for this study.

Considering feasibility and blinded validity, conducting this study as a double-blind randomized controlled trial is challenging. The total number of sessions in this study is 40 and the duration of maintenance therapy is 12 months, which is considered to be a significant burden for the participants assigned to sham stimulation owing to a large number of sessions and the long duration of the study. Moreover, since all the participants in this study received acute rTMS therapy before entering the maintenance rTMS therapy period, patients may be able to distinguish between real and sham stimulation, which would reduce the blinded validity of the study. Thus, an open-label, nonrandomized study design will be employed in this study.

As mentioned before, the risk of bias owing to the lack of blinding and randomization is a limitation of this study. For example, patients who are favorable to rTMS therapy may be more likely to be assigned to the maintenance rTMS group, resulting in a larger placebo effect and overestimating the effect of maintenance rTMS therapy. We plan to address this point in two ways. The first is to adjust for the background factors by performing IPTW analysis as a sensitivity analysis and attempting to increase the two groups' comparability. However, risk of bias remains because unmeasured confounding cannot be eliminated by an IPTW analysis. Second, the long-term outcome of post-marketing surveillance of the Nerurostar TMS device in Japan will be used as a historical control. Comparing the maintenance therapy group with both the control group in this study and the historical control group may reduce the possibility of overestimating the effect of treatment.

If maintenance rTMS is shown to be effective in preventing relapse/recurrence of depression in this study, the treatment options for patients who have had difficulty maintaining remission with antidepressants and/or psychotherapy will be expanded. Since rTMS is a developing technology, there is no evidence that the current stimulation protocol is the best for treating patients with MDD. The search for optimal protocols for rTMS maintenance therapy and updates to accommodate new rTMS stimulation methods, which are being improved daily, should be continued in future studies.

### Trial status

Ongoing.

## Supplementary Information

**Additional file 1.**

## Data Availability

The datasets generated and/or analyzed during this study will not be publicly available but will be available from the corresponding author upon reasonable request.

## Abbreviations

rTMS: Repetitive transcranial magnetic stimulation
MDD: Major depressive disorder
HAMD-17: Hamilton Rating Scale for Depression 17 item
MADRS: Montgomery–Åsberg Depression Rating Scale
QIDS: Quick Inventory of Depressive Symptomatology
PHQ-9: Patient Health Questionnaire-9
DSM-5: Diagnostic and Statistical Manual of Mental Disorders, 5th edition
IPTW: Inverse probability of treatment weighting
