# Supplementary material for: Maintenance repetitive transcranial magnetic stimulation (rTMS) therapy for treatment-resistant depression: a study protocol of a multisite, prospective, non-randomized longitudinal study
Source: BMC Psychiatry. 2023 Jun 16;23:437. doi: 10.1186/s12888-023-04944-0 (PMC10273734; doi:10.1186/s12888-023-04944-0)
Supplement: Supplementary file 1 — Additional file 1. [file 12888_2023_4944_MOESM1_ESM.docx]

**Supplementary file1**

## Sponsor and principal investigator

Shinsuke Kito

Director, Department of Psychiatry, National Center of Neurology and Psychiatry Hospital

4-1-1 Ogawahigashimachi, Kodaira-shi, Tokyo

Telephone: +81 42-341-2711

Web: <https://www.ncnp.go.jp/en/>

Mail: [kito@ncnp.go.jp](mailto:kito@ncnp.go.jp)

**・Composition, roles, and responsibilities of the coordinating center**

| **Affiliation / Titles** | **Name** | **Role in Research** |
| --- | --- | --- |
| Head of Biostatistical Analysis Office, Department of Clinical Data Science, Clinical Research and Education Premotion Division, National Center of Neurology and Psychiatry | Mari Oba | Head of statistical analysis |
| Head of Business Development Group, Satt Corporation | Kosaku Kawada | Monitoring manager |
| Head of Data Management Office, Department of Clinical Data Science, Clinical Research and Education Premotion Division, National Center of Neurology and Psychiatry | Kenji Hatano | Head of data management |
| Member of Audit Office, National Center of Neurology and Psychiatry | Naoki Tsutsuzumi | Audit manager |
| Director, Department of Clinical Data Science, Clinical Research and Education Premotion Division, National Center of Neurology and Psychiatry | Hideki Oi | Protocol development advisor |
| National Center of Neurology and Psychiatry Hospital, Department of Psychiatry, Chief physician | Takamasa Noda | Trial manager |

## Steering committee

| **Affiliation / Titles** | **Name** | **Roles and responsibilities** |
| --- | --- | --- |
| Director, National Center of Neurology and Psychiatry Hospital, Department of Psychiatry | Shinsuke Kito | Committee chairman, trial sponsor and principal investigator |
| Chief physician, National Center of Neurology and Psychiatry Hospital, Department of Psychiatry | Takamasa Noda | Committee member, trial manager |
| Physician, National Center of Neurology and Psychiatry Hospital | Daisuke Hayashi | Committee member |
| Researcher, National Center of Neurology and Psychiatry Hospital | Junko Matsuo | Committee member |
| Chief physician, The Jikei University Hospital | Yuki Matsuda | Committee member |
| Physician, The Jikei University Hospital | Ryuichi Yamazaki | Committee member |

**・The committee for efficacy and safety assessment** **(endpoint adjudication committee)**

| **Affiliation / Titles** | **Name** | **Roles and responsibilities** |
| --- | --- | --- |
| Deputy director, Showa University Medical Institute of Developmental Disabilities Research | Motoaki Nakamura | Committee chairman |
| Professor, Department of Rehabilitation, International University of Health and Welfare | Wataru Kakuda | Committee member |
| Director, Hibiya Industrial Physician Office | Takashi Hasegawa | Committee member |

・**The list of participated institutions**

| **Institution** | **Address** |
| --- | --- |
| National Center of Neurology and Psychiatry Hospital | 4-1-1 Ogawahigashi, Kodaira-shi, Tokyo, 187-8551, Japan |
| Jikei University Hospital | 3-25-8 Nishi-Shinbashi, Minato-ku, Tokyo, 105-8461, Japan |
| Kanagawa Psychiatric Center | 2-5-1 Serigaya, Konan-ku, Yokohama-shi, Kanagawa, 233-0006, Japan |
| Saga University Hospital | 5-1-1 Nabeshima, Saga-shi, Saga, 849-0937, Japan |
| St. Luke's Hospital | 1012 Tsufuku Honmachi, Kurume-shi, Fukuoka, 830-0047, Japan |
| Osaka Medical and Pharmaceutical University Hospital | 2-7 Daigakucho, Takatsuki-shi, Osaka, 569-8686, Japan |
| Kumamoto University Hospital | 1-1-1 Honjo, Chuo-ku, Kumamoto-shi, Kumamoto, 860-8556, Japan |
| Fujita Health University Hospital | 1-98 Dengakugakubo, Kutsukake-cho, Toyoake-shi, Aichi, 470-1192, Japan |
| Kansai Medical University Hospital | 2-3-1 Shinmachi, Hirakata-city, Osaka, 573-1191, Japan |
| Saitama Medical University Hospital | 1397-1 Yamane, Hidaka-shi, Saitama, 350-1298, Japan |
| Yuge Hospital | 5-12-25 Yuge, Kita-ku, Kumamoto-shi, Kumamoto, 861-8002, Japan |
| Miyakonojo Shinsei Hospital | 3782 Shibita-cho, Miyakonojo-shi, Miyazaki, 885-0093, Japan |
| Asakayama Hospital | 3-3-16 Imaikemachi, Sakai-ku, Sakai-shi, Osaka, 590-0018, Japan |
